# From patient-derived tumor organoids to personalized cancer therapy: advancing treatment for advanced solid tumors

**DOI:** 10.3389/fonc.2026.1880986

**Published:** 2026-07-20

**Authors:** Gang Wang, Zhibin Tan, Wenting Jia, Yuanyuan Liu, Huan Wang, Wenbo Sun, Weiwei Sun, Lu Meng, Yudong Zhang, Ning Liu, Feng Li, Hongchao Dong

**Affiliations:** 1 Department of Chemoradiation, North China University of Science and Technology Affiliated Hospital, Tangshan, Hebei, China; 2 Department of Pain, North China University of Science and Technology Affiliated Hospital, Tangshan, Hebei, China; 3 Central Laboratory, Shanxi Province Cancer Hospital/Shanxi Hospital Affiliated to Cancer Hospital, Chinese Academy of Medical Sciences/Cancer Hospital Affiliated to Shanxi Medical University, Taiyuan, China; 4 Department of Thoracic Surgery, Tangshan Gongren Hospital, Tangshan, Hebei, China

**Keywords:** advanced malignancies, culture strategies, patient-derived tumor organoids, precision medicine, treatment response

## Abstract

**Background:**

Cancer remains a major global public health burden, with high morbidity and mortality. Despite advances in treatment strategies, many patients with advanced solid tumors face treatment resistance and limited therapeutic options. Patient-derived tumor organoids (PDTOs) have emerged as promising preclinical models for personalized medicine, but their clinical translational potential in guiding individualized treatment for advanced solid tumors requires further validation.

**Methods:**

The sample data of 164 solid tumors used for establishing organoids were retrospectively analyzed, and a clinical cohort involving 23 patients with advanced solid tumors was used to validate the feasibility of PDTOs in predicting the treatment response. The concordance between PDTO drug responses and clinical outcomes was evaluated using Cohen’s Kappa test.

**Results:**

The overall establishment success rate of organoids reached 91.5%, with the rates ranging from 90.6% to 92.6% across different cancer types. Histopathological analysis confirmed that PDTOs partially recapitulated the histopathological features and proliferative activity of matched original tumors. PDTO-based drug sensitivity testing showed good concordance with clinical treatment outcomes of patients with advanced solid tumors, yielding an overall accuracy of 85.0%, a sensitivity of 86.7%, a specificity of 80.0%. Clinical case studies further highlighted the unique value of PDTOs in overcoming multidrug resistance in advanced solid tumors.

**Conclusions:**

This study demonstrates that PDTOs exhibit favorable potential for predicting treatment responses in patients with advanced solid tumors, and may serve as a promising auxiliary companion diagnostic tool to inform personalized cancer therapy and assist in evaluating multidrug resistance for advanced solid tumors. Future large-scale, multicenter studies with stratified subgroup validations are warranted to further verify the clinical translational value of PDTOs in individualized tumor treatment.

## 1 Introduction

As a major social and public health problem, cancer is globally responsible for almost 16.8% deaths and 22.8% deaths from noncommunicable diseases. According to global cancer statistics 2022, there were nearly 20 million new cases of cancer and 9.7 million deaths from cancer in 2022 (1). With the popularization of cancer-screening methods, utilization of multidisciplinary team approaches and implementation of precision oncology, the 5-year survival rate of cancer patients in China has been improved significantly over the past few decades (2). However, substantial clinical challenges remain unresolved. Clinically, the efficacy of antitumor therapies varies greatly among individual patients with the same tumor type and pathological stage (3, 4). Even when actionable genetic mutations are identified via next-generation sequencing to guide targeted drug selection, a large proportion of patients still fail to respond to standardized treatments or develop drug resistance rapidly.

The core bottleneck restricting individualized cancer treatment is the lack of reliable and clinically applicable *in vitro* models for drug sensitivity prediction. Although next-generation sequencing offers high-throughput profiling of the cancer genomic landscape to inform individualized treatment strategies (5–7), it faces considerable challenges in clinical translation (8, 9). Genomic data only capture tumor genetic alterations but fail to recapitulate the intact spatial architecture, cellular heterogeneity and complex biological features of primary tumors, making it difficult to translate massive genomic data into clinically actionable guidance. Accordingly, patients harboring targetable mutations often show poor responses to matched targeted therapies (10), which undermines the accuracy of drug response prediction and reduces the precision of personalized treatment. This unmet clinical gap greatly limits the overall efficacy of individualized cancer therapy.

Patient-derived tumor organoids (PDTOs), as three-dimensional (3D) *in vitro* tumor culture models, can not only retain the biological behaviors and function of the original tumor but also recapitulate the intricate spatial architecture and cellular heterogeneity (11). Based on the organoid drug sensitivity analysis, the optimal treatment regimen can be identified at the individual level, thereby guiding treatment decisions and improving patient outcomes (12). In the present study, we described the key culture strategies for establishing the organoids from solid tumors, and explored the feasibility of PDTOs in guiding the individualized treatment of patients with advanced solid tumors at the patient-specific level.

## 2 Materials and methods

### 2.1 Sample source

Between October 2021 and March 2023, the sample data of 164 solid tumors used for establishing organoids from the database of Kingbio Medical Co. Ltd. (Chongqing, China) were retrospectively analyzed. Specific cancer types and their corresponding number of cases are described in **Table 1**. Regarding organoid culture and research, informed consent was obtained prior to acquisition of samples. In addition, a clinical cohort of 23 patients with pathologically confirmed advanced solid tumors was enrolled to validate the predictive performance of PDTOs for clinical treatment responses. Eligibility criteria included successful organoid establishment and evaluable tumor responses according to Response Evaluation Criteria in Solid Tumors (RECIST) version 1.1. Patients with incomplete follow-up data, non-measurable lesions or discontinued anti-cancer treatment were excluded. All enrolled participants provided written informed consent prior to sample collection. This study was approved by the Institutional Review Board of North China University of Science and Technology Affiliated Hospital (Approval No.: 20241226011), and was conducted in accordance with the principles of Declaration of Helsinki.

**Table 1:** The success rates of organoid establishment across different cancer types.

Cancer types	Organoids/total samples	Success rates	Different sources					
			Organoids/surgical samples	Success rates	Organoids/biopsies	Success rates	Organoids/malignant effusion samples	Success rates
Pancreatic cancer	32/35	91.4%	18/19	94.74%	8/10	80.00%	6/6	100.00%
Gastric cancer	33/36	91.7%	19/21	90.48%	5/6	83.33%	9/9	100.00%
Breast cancer	29/32	90.6%	13/15	86.67%	11/12	91.67%	5/5	100.00%
Cholangiocarcinoma	31/34	91.2%	17/18	94.44%	8/9	88.89%	6/7	85.71%
Lung cancer	25/27	92.6%	8/8	100.00%	9/10	90.00%	8/9	88.89%
Overall	150/164	91.5%	75/81	92.60%	41/47	87.23%	34/36	94.44%

Success rates were calculated as: Success rates = number of successfully established organoids/total cultured samples × 100%.

### 2.2 Organoid culture

Based on the protocols provided by Kingbio Medical Co. Ltd. (Chongqing, China), PDTOs were cultured and drug sensitivity testing was strictly performed. Specifically, the tumor tissues from surgical resection or biopsy were minced into approximately 1 mm³ fragments in cold Advanced DMEM/F12 medium supplemented with antibiotics after removing necrotic and fibrotic tissues, and then were dissociated enzymatically at 37 °C for 20–30 min with gentle agitation. The digested mixture was sequentially filtered through 70 µm and 40 µm strainers, and centrifuged at 300×g for 5 min to collect cell clusters. Subsequently, the resulting small clusters were embedded in Matrigel (Corning) and plated in multi-well plates. Once Matrigel was solidified, the organoid culture medium was added to each well and changed every 2–3 days. The culture was performed at 37 °C, 5% CO_2_. At the time of dense, the organoids were passaged every 7–14 days. Notably, the samples from pleural effusion or ascites were directedly centrifugated at 300×g for 5 min after acquisition, without digestion. Successful organoid establishment was defined by the following criteria: (1) formation of typical 3D organoid structures in extracellular matrix; (2) sustained growth during primary culture; (3) ability to be maintained for at least two passages; (4) absence of bacterial, fungal, or mycoplasma contamination; (5) preservation of tumor-like morphology by histological assessment when available; and (6) generation of sufficient organoid material for downstream drug sensitivity testing.

After completion of organoid dissociation, a series of operations, such as dehydration, transparency, waxdip and embedding, were conducted. The paraffin-embedded organoids and tissues were sectioned for hematoxylin and eosin (H&E) staining to compare their histological architecture, glandular/tumor-like structures, and cellular morphology. Ki67 immunohistochemistry (IHC) was used to evaluate the proliferative activity of the organoids. Briefly, paraffin sections were deparaffinized, rehydrated, subjected to antigen retrieval, blocked, incubated with anti-Ki67 primary antibody overnight at 4 °C, followed by incubation with the corresponding secondary antibody before visualization.

### 2.3 Drug sensitivity testing

Organoids were dissociated into small fragments or single-cell suspensions according to their growth status, and approximately 500 viable organoid-derived cells were seeded into each well of a 384-well plate. Following overnight recovery, cells were exposed to the indicated drugs prepared as 7-point serial dilutions, mainly including chemotherapeutic agents, targeted agents and monoclonal antibodies. Drug concentration ranges were determined based on the upper and lower limits of clinically relevant blood drug concentrations. Serial dilution gradients were set separately for single-agent regimens and combination regimens. Single agents were tested over a molar concentration range of 1×10^-10^ M to 1×10^-4^ M, while combination treatments covered concentrations spanning 0.00001 Cmax to 10 Cmax (**Supplementary Table 1**). Vehicle-treated wells with an equal volume of solvent were included as negative controls, and at least three biological replicates were established for each treatment condition. The final concentration of vehicle was maintained at 0.1% (v/v) across all culture wells.

After 72 h of drug treatment, organoid viability was measured using CellTiter-Glo 2.0 according to the manufacturer’s instructions, and luminescence was detected using a microplate reader. Relative viability was calculated by normalizing the luminescence signal of drug-treated wells to that of vehicle-treated control wells. According to the quantification of the inhibitory effects of drugs on tumor organoid growth, a five-category classification was utilized for evaluating the drug efficacy, including highly sensitive (80%-100%), sensitive (60%-80%), intermediate sensitive (40%-60%), lowly sensitive (20%-40%) and resistant (0%-20%) types (13, 14).

### 2.4 Assessment of clinical outcomes

Clinical treatment responses were evaluated strictly in accordance with the RECIST version 1.1. All patients underwent contrast-enhanced computed tomography (CT) or magnetic resonance imaging (MRI) to identify tumor lesions and dynamically monitor treatment responses. Imaging evaluation was performed as follows: baseline imaging was acquired within one week before the initiation of anti-tumor treatment to confirm target and non-target lesions; routine follow-up imaging was scheduled every 6–8 weeks during treatment, with additional unscheduled imaging examinations conducted whenever clinical symptoms or laboratory abnormalities suggested potential disease progression.

The patients were considered to have a good response if complete response (CR) or partial response (PR) was achieved. For the patients with incurable locally advanced or metastatic solid tumors, the major purpose of the clinical treatment was to improve the prognosis of patients and prolong the survival, thus stable disease (SD) was acceptable (15). The toxicity of drug treatment was evaluated using Common Terminology Criteria for Adverse Event version 4.0.

### 2.5 Statistical analysis

For baseline clinical characteristics, continuous variables including treatment cycles were analyzed using descriptive statistics, and the results were presented as the median, minimum and maximum values. Cohen’s Kappa test was performed to analyze the concordance between PDTO-based drug sensitivity testing and clinical treatment outcomes of patients. In this study, patients with PR or SD were considered to have a good response, while those with progressive disease (PD) were regarded to have a poor response (15). The numbers of true positives, true negatives, false positives, and false negatives were calculated, followed by the determination of sensitivity, specificity, accuracy, and Kappa coefficient to comprehensively assess the diagnostic performance. The criteria for evaluating the Kappa coefficient were defined as follows: poor (<0.20), fair (0.20-0.40), moderate (0.40-0.60), good (0.60-0.80) and very good (>0.80%) concordance (16).

## 3 Results

### 3.1 Establishment and characterization of a living organoid biobank

**Figure 1:**
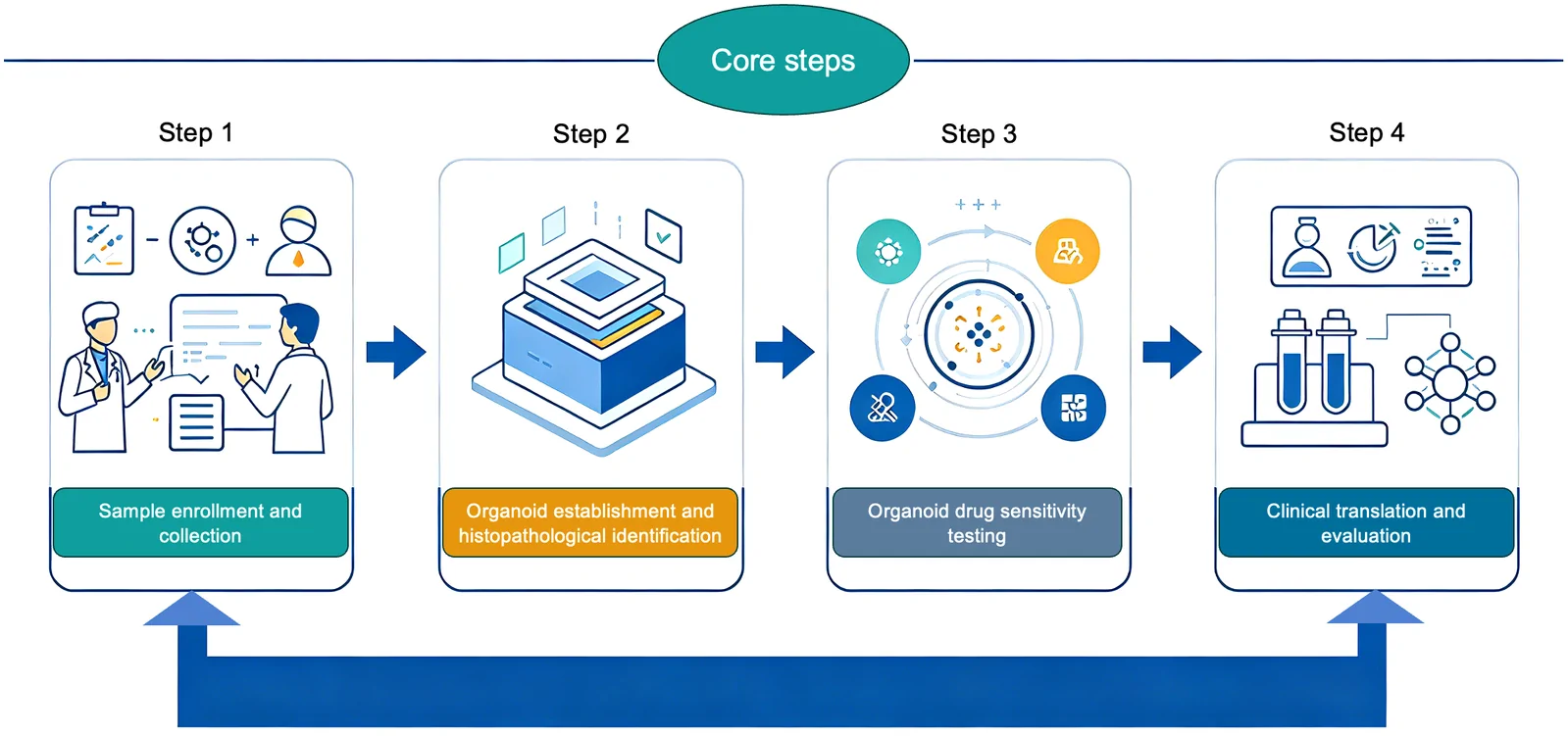
The schematic diagram illustrating the overall workflow of PDTO establishment, drug sensitivity testing, and clinical translational validation.

**Figure 2:**
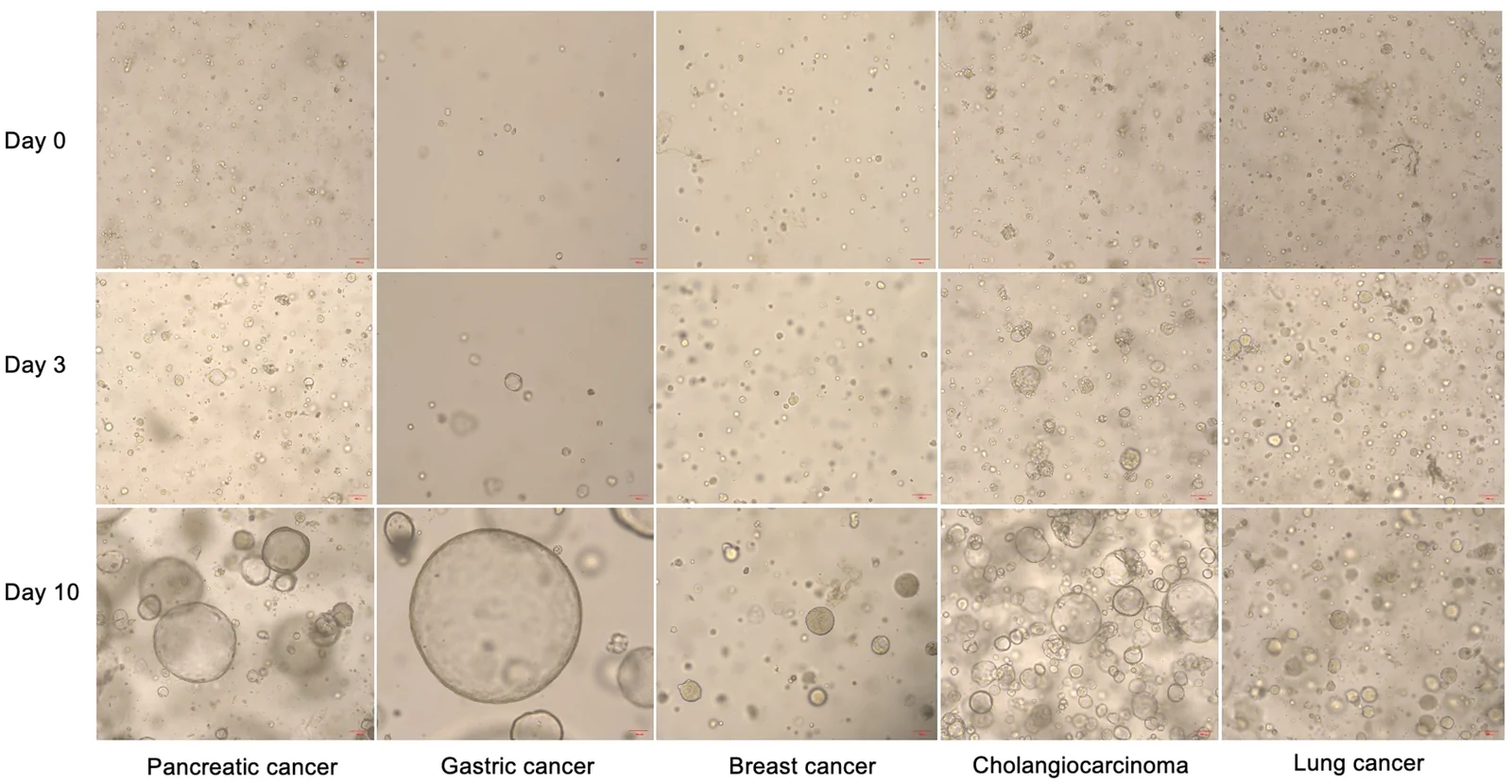
The growth status of organoids established from different cancer types at consecutive culture time points. Representative bright-field microscopy images display the dynamic growth status and morphological changes of organoids at different culture stages. All images were uniformly captured at a magnification of ×100. Notably, these organoid samples are from the 164 biobank tumor specimens used to assess organoid establishment rates, which are distinct from the 23-patient clinical validation cohort.

A schematic diagram (**Figure 1**) was constructed to depict the overall technical procedures and clinical translation implemented in this study. Between October 2021 and March 2023, 164 solid tumor samples from Kingbio database were selected for organoid culture, including pancreatic cancer (n=35), gastric cancer (n=36), breast cancer (n=32), cholangiocarcinoma (n=34) and lung cancer (n=27). Briefly, freshly obtained tumor tissues were mechanically minced and enzymatically digested, followed by filtration, centrifugation, and embedding in extracellular matrix. The embedded cells/fragments were cultured in organoid medium under standard incubator conditions, and the medium was refreshed regularly. Organoid growth was monitored by bright-field microscopy. Based on the organoid establishment criteria, the success rates of organoid establishment were found to range from 90.6% to 92.6% across different cancer types, with the overall success rate reaching up to 91.5% (**Table 1**). The growth status of organoids from different cancer types at consecutive culture time points is presented in **Figure 2**.

For each cancer type, the success rates of generating organoids from different sources were slightly different, and they were all higher than 80.00% (**Table 1**). Considering that the 91.5% overall establishment rate was markedly higher than that reported in previous studies (15, 17, 18), we further clarified the core cause of elevated culture efficiency, and confirmed that the elevated culture efficiency is mainly attributed to our source-targeted optimized culture strategies, rather than sample selection bias. To improve the culture success rates, targeted optimizations of culture strategies was implemented in the organoid platform based on the biological characteristics of samples from different sources. For tissue samples, refined primary cell isolation and processing procedures were adopted to maximize the preservation of cell viability and heterogeneity. For malignant effusion samples with low cell abundance, efficient tumor cell enrichment and specialized culture protocols were employed, which significantly improved the success rate of organoid establishment. Notably, for conventionally hard-to-culture tumor types such as pancreatic cancer, this platform effectively mitigated the damage of endogenous digestive enzymes to cell viability through a unique culture system with proprietary patented preservation solution formula which can effectively maintain cellular homeostasis, and promoted spontaneous cell aggregation and survival, thereby achieving a marked increase in organoid formation. Additionally, to ensure the quality and reliability of the established organoid models, this platform formulated a standardized operating procedure and implemented a rigorous quality control system throughout the entire process, comprising various steps such as cell viability detection, microbial contamination screening, and validation of key phenotypes.

### 3.2 Histopathological identification of PDTOs from different cancer types

**Figure 3:**
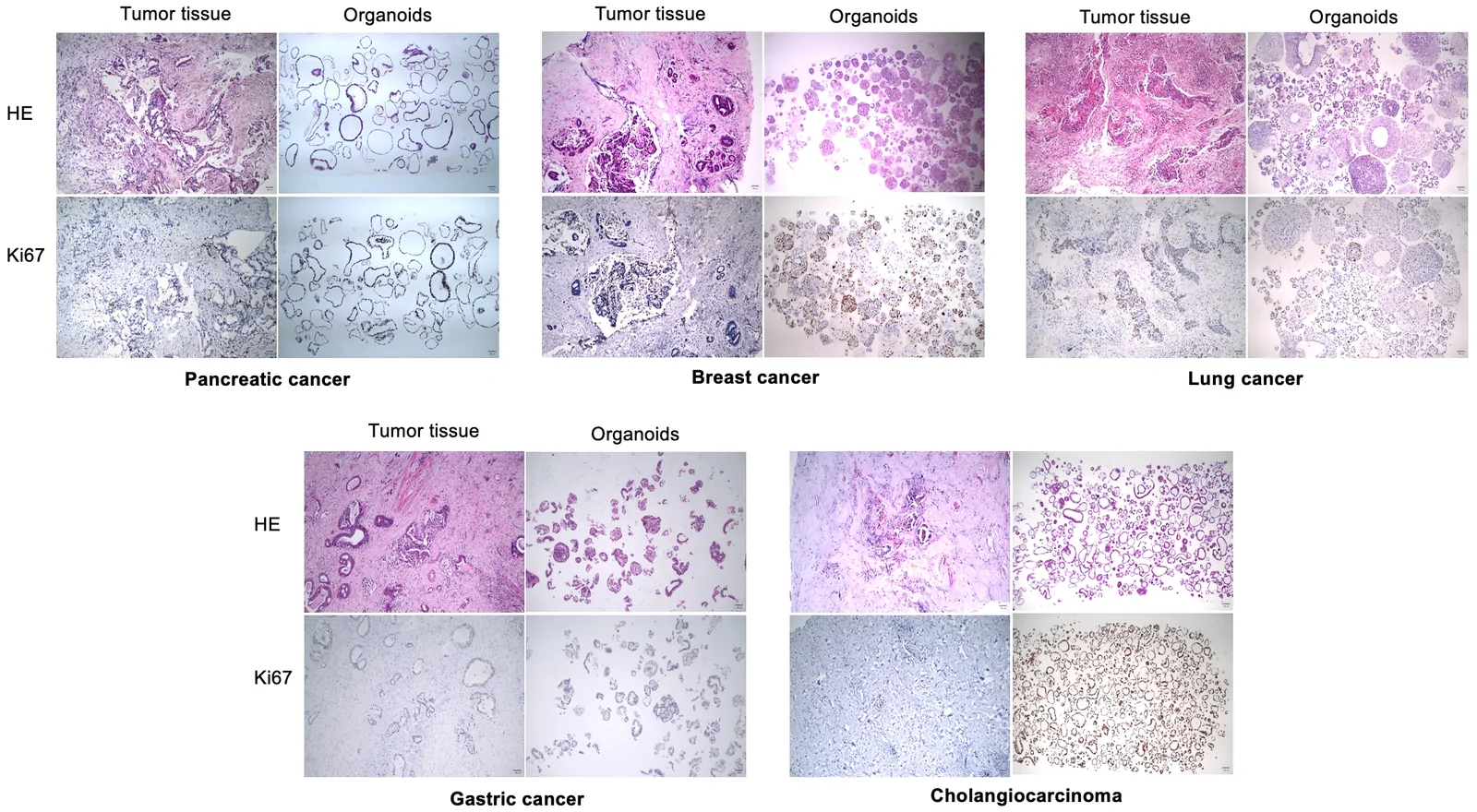
Histopathological consistency between primary tumor tissues and corresponding organoids. Hematoxylin and Eosin (H&E) staining and Ki-67 immunohistochemical staining were performed to compare histological structures and tumor proliferative activity. All microscopic images were acquired at ×100 magnification. Notably, these organoid samples are from the 164 biobank tumor specimens used to assess organoid establishment rates, which are distinct from the 23-patient clinical validation cohort.

H&E and IHC staining was both performed to validate the consistency of PDTOs with corresponding tumor tissues. It could be observed a relatively homogeneous appearance in the organoids from different cancer types, such as pancreatic cancer, gastric cancer, breast cancer, cholangiocarcinoma and lung cancer (**Figure 3**). Meanwhile, these organoids exhibited consistent Ki67 expression patterns relative to the original tumor tissues. These results indicate that the organoid models partially recapitulate the histopathological characteristics and proliferative activity of primary tumors.

### 3.3 Concordance between PDTO-based drug sensitivity testing and clinical treatment outcomes

**Figure 4:**
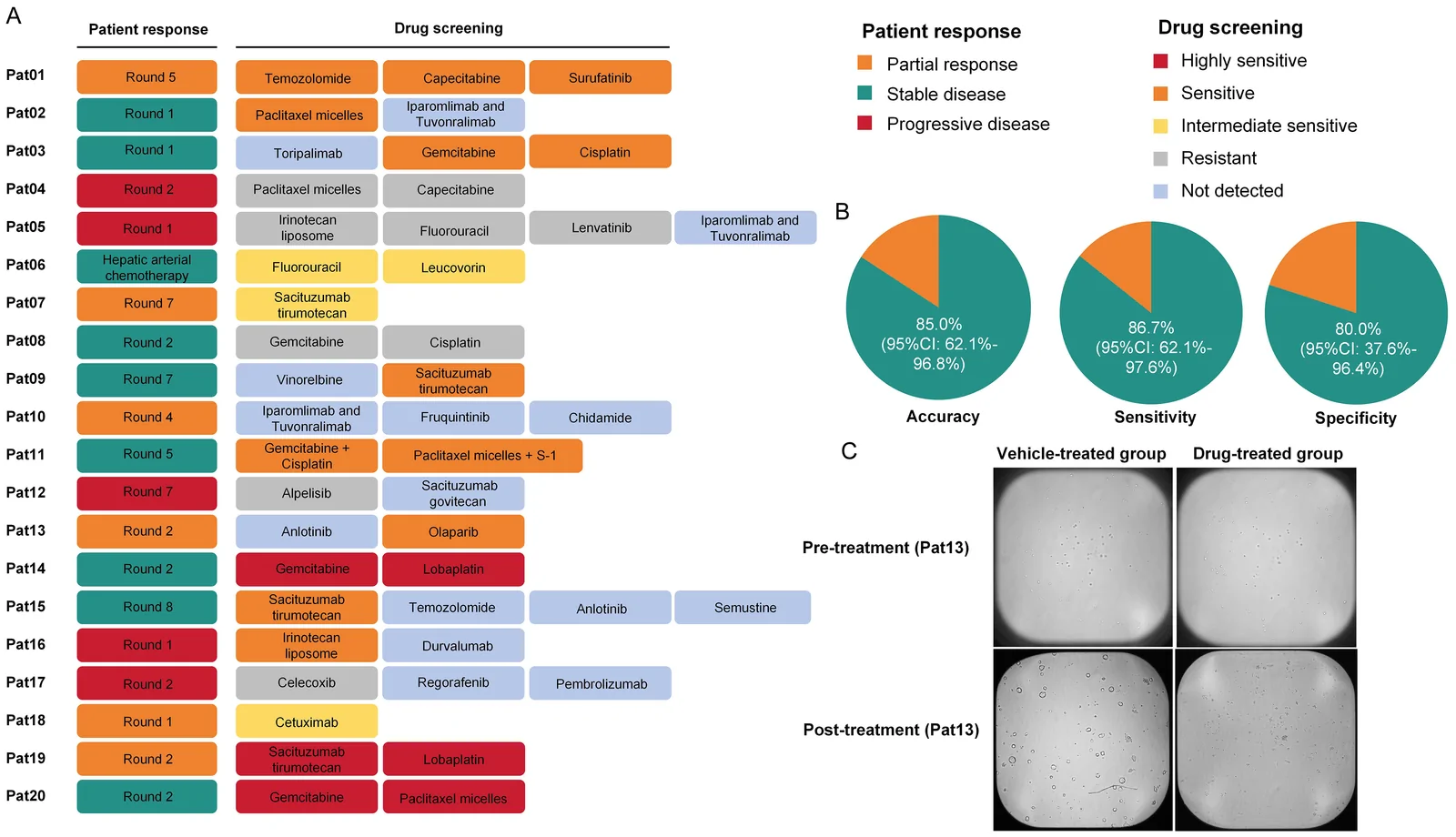
Concordance between organoid drug screening and clinical treatment outcomes. Totally 20 patients with advanced solid tumors were enrolled to systematically compare organoid drug sensitivity results and clinical treatment responses. **(A)** The heatmap shows the individual correspondence between clinical treatment outcomes and matched organoid drug screening results across all enrolled patients. **(B)** Pie charts quantitatively present the core statistical indicators of the organoid prediction model, including sensitivity, specificity, and overall accuracy. **(C)** Bright-field imaging of PDTOs derived from Pat13 to compare organoid morphology with or without doxorubicin exposure. All images were acquired from single wells of a 384-well plate on a Celigo imaging cytometer equipped with a 10× objective lens. The vehicle-treated group was cultured with an equivalent volume of vehicle only, while doxorubicin was administered to the treatment group. Representative images reveal intact morphology and clonal proliferation in vehicle-treated organoids; conversely, doxorubicin-treated organoids present evident shrinkage, fragmentation and diminished volume in response to drug sensitivity.

To verify whether PDTO-based drug sensitivity testing could mirror the previous drug responses ofpatients, a cohort of 23 patients with clear retrospective data was followed up, and their representative bright-field organoid images were presented in **Supplementary Figure 1**. Notably, 3 patients were unable to receive anti-tumor treatment after drug sensitivity testing due to poor general condition, thus they abandoned the treatment. Finally, 20 patients who received the precision treatment based on the PDTO drug sensitivity testing and were assessed radiologically were included in the concordance analysis (**Table 2**). The median organoid drug testing turnaround time for this cohort was 28 days, with patients receiving a median of 2 treatment cycles (range: 1-8). The clinical treatment outcomes of these 20 patients and their corresponding drug screening results are depicted in **Figure 4A**.

**Table 2:** The baseline information and treatment outcomes of 20 patients with advanced solid tumors included in the concordance analysis.

Identifiers	Age/years	Gender	Tumor types	Tumor stage	ECOG score	Median turnaround time/days	Organoid establishment status	Treatment regimes	Organoid drug sensitivity results	Treatment cycles	Outcomes
Pat01	64	Male	Thymic neuroendocrine tumor	Stage IV	1	28	Successfully established	Temozolomide + Capecitabine + Surufatinib	Temozolomide + Capecitabine + Surufatinib (sensitive)	5	PR
Pat02	47	Male	Pancreatic cancer	Stage IV	2	19	Successfully established	Paclitaxel micelles + Iparomlimab and Tuvonralimab	Paclitaxel micelles (sensitive)	1	SD
Pat03	47	Male	Hilar cholangiocarcinoma	Stage IV	2	28	Successfully established	Toripalimab + Gemcitabine + Cisplatin	Gemcitabine + Cisplatin (sensitive)	1	SD
Pat04	72	Female	Breast cancer	Stage IV	3	28	Successfully established	Paclitaxel micelles + Capecitabine	Paclitaxel micelles + Capecitabine (resistant)	2	PD
Pat05	42	Female	Cholangiocarcinoma	Stage IV	1	33	Successfully established	Irinotecan liposome + Fluorouracil + Lenvatinib + Iparomlimab and Tuvonralimab	Irinotecan liposome + Fluorouracil + Lenvatinib (resistant)	1	PD
Pat06	66	Male	Colorectal cancer	Stage IV	1	36	Successfully established	Fluorouracil + Leucovorin	Fluorouracil + Leucovorin (intermediate sensitive)	Hepatic arterial chemotherapy	SD
Pat07	58	Female	Gallbladder carcinoma	Stage IV	1	25	Successfully established	Sacituzumab Tirumotecan	Sacituzumab Tirumotecan (intermediate sensitive)	7	PR
Pat08	73	Male	Cholangiocarcinoma	Stage IV	2	35	Successfully established	Gemcitabine + Cisplatin	Gemcitabine + Cisplatin (resistant)	2	SD
Pat09	62	Female	Lung adenocarcinoma	Stage IV	1	35	Successfully established	Sacituzumab Tirumotecan	Sacituzumab Tirumotecan (sensitive)	7	SD
Pat10	63	Male	Colon cancer	Stage IV	1	35	Successfully established	Iparomlimab and Tuvonralimab + Fruquintinib + Chidamide	None detected	4	PR
Pat11	60	Male	Cholangiocarcinoma	Stage IV	2	27	Successfully established	Gemcitabine + Cisplatin (2 cycles) and Paclitaxel micelles + S-1 (3 cycles)	Gemcitabine + Cisplatin (sensitive); Paclitaxel micelles + S-1 (sensitive)	5	SD
Pat12	45	Female	Breast cancer	Stage IV	2	28	Successfully established	Alpelisib + Sacituzumab govitecan	Alpelisib (resistant)	7	PD
Pat13	76	Female	Ovarian cancer	Stage IV	2	28	Successfully established	Anlotinib + Olaparib	Olaparib (sensitive)	2	PR
Pat14	70	Male	Pancreatic cancer	Stage IV	2	27	Successfully established	Gemcitabine + Lobaplatin	Gemcitabine + Lobaplatin (highly sensitive)	2	SD
Pat15	64	Male	Lung cancer	Stage IV	1	26	Successfully established	Sacituzumab govitecan + Temozolomide + Anlotinib + Semustine	Sacituzumab govitecan (sensitive)	8	SD
Pat16	64	Male	Small cell lung cancer	Stage IV	1	28	Successfully established	Irinotecan liposome + Durvalumab	Irinotecan liposome (sensitive)	1	PD
Pat17	69	Male	Colorectal cancer	Stage IV	3	28	Successfully established	Celecoxib + Regorafenib + Pembrolizumab	Celecoxib (resistant)	2	PD
Pat18	51	Female	Pancreatic cancer	Stage IV	2	24	Successfully established	Cetuximab	Cetuximab (intermediate sensitive)	1	PR
Pat19	59	Female	Intrahepatic cholangiocarcinoma	Stage IV	1	35	Successfully established	Sacituzumab Tirumotecan + Lobaplatin	Sacituzumab Tirumotecan + Lobaplatin (highly sensitive)	2	PR
Pat20	66	Male	Pancreatic cancer	Stage IV	2	26	Successfully established	Gemcitabine + Paclitaxel micelles	Gemcitabine + Paclitaxel micelles (highly sensitive)	2	SD

Tumor stage is based on AJCC, 8^th^ edition.

PR, partial response; SD, stable disease; PD, progressive disease; ECOG, Eastern Cooperative Oncology Group.

It should be noted that 13 patients (13/15, 86.7%) receiving the treatments, which comprised at least one drug that was identified intermediate sensitive, sensitive and highly sensitive by the PDTOs, achieved PR or SD. In contrast, among the 5 patients with PD, 4 (4/5, 80.00%) received treatment regimens containing drugs that were identified as resistant in PDTO-based drug sensitivity testing and subsequently developed PD. These findings demonstrate a good concordance between PDTO-based drug sensitivity testing results and clinical treatment outcomes (Kappa coefficient = 0.625; 95%CI: 0.302-0.948), with the testing yielding an overall accuracy of 85.0% (95%CI: 62.1%-96.8%), a sensitivity of 86.7% (95%CI: 62.1%-97.6%), and a specificity of 80.0% (95%CI: 37.6%-96.4%) (**Figure 4B**). The typical bright-field imaging of PDTOs derived from Pat13 is shown in **Figure 4C** to compare organoid morphology with or without doxorubicin exposure.

### 3.4 Prediction of clinical response based on the PDTO drug treatment

A key objective of this platform is its capacity to identify uniquely sensitive drugs for each patient. In the present study, our focus was primarily directed toward advanced solid tumors exhibiting multidrug resistance.

**Figure 5:**
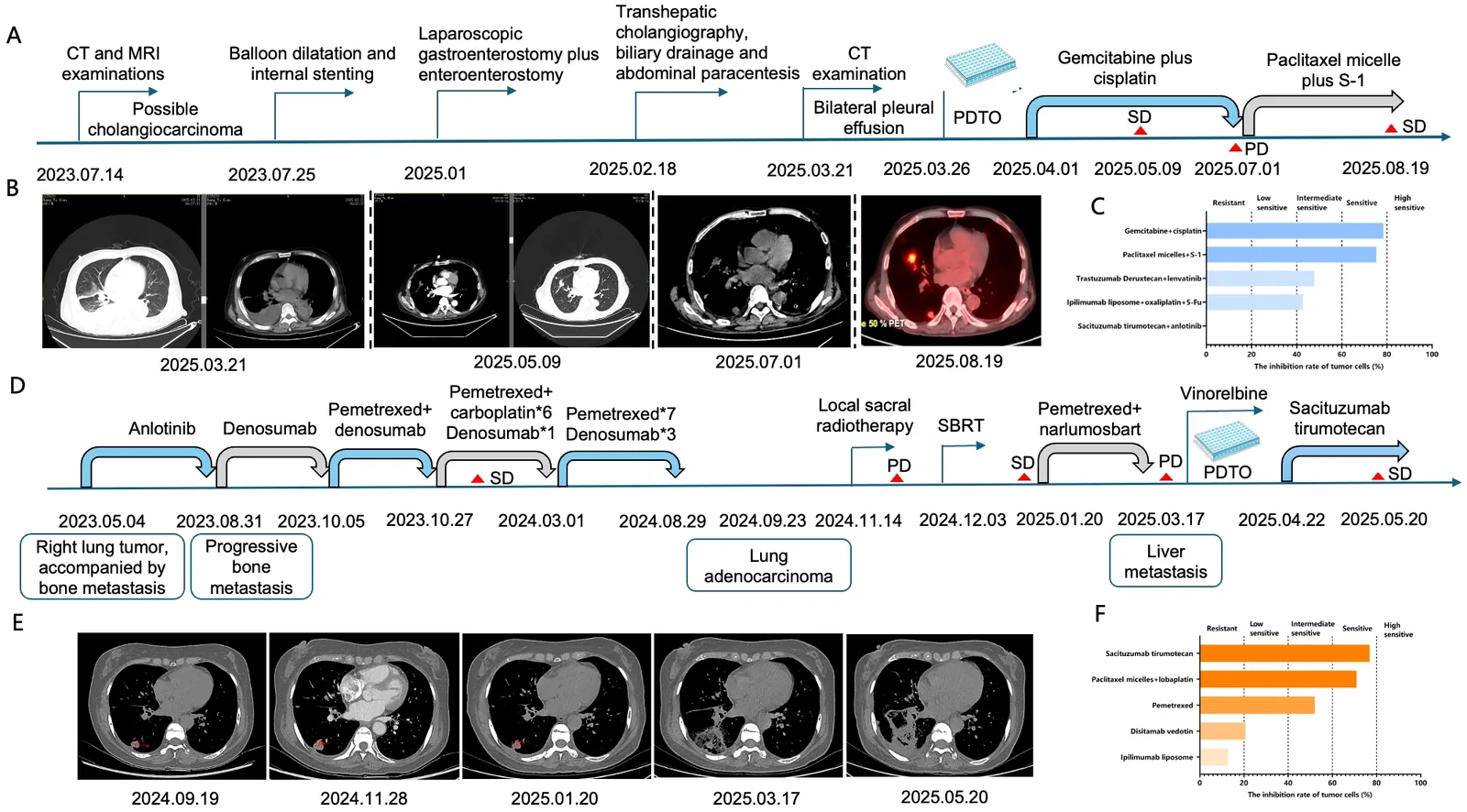
Case verification of individualized predictive efficacy of organoid drug sensitivity testing in two representative patients with advanced solid tumors. Clinical diagnosis and treatment timeline **(A)**, dynamic radiological changes during treatment **(B)**, and corresponding drug sensitivity testing results **(C)** in Pat11 with advanced cholangiocarcinoma. Clinical diagnosis and treatment course **(D)**, serial radiological variations during treatment **(E)**, and matched organoid drug screening results **(F)** in Pat09 with advanced lung adenocarcinoma.

Pat11 was diagnosed with stage IV cholangiocarcinoma, complicated by multi-site lymph node metastasis, bone metastasis, lung metastasis, and malignant pleural effusion. On July 25, 2023, the patient underwent balloon dilatation and internal stenting for biliary obstruction, followed by 6 cycles of postoperative chemotherapy (**Figure 5A**). A re-examination in January 2025 indicated tumor progression, and then laparoscopic gastroenterostomy plus enteroenterostomy were performed. On February 18, 2025, emergency percutaneous transhepatic cholangiography, biliary drainage and abdominal paracentesis catheterization were performed. A CT scan on March 21, 2025, revealed bilateral pleural effusion (**Figure 5B**). With the patient’s consent, right pleural effusion was collected for organoid culture. On April 1, 2025, the patient was administered gemcitabine combined with cisplatin, along with intrathoracic injection of lobaplatin. Considering that the patient was sensitive to gemcitabine plus cisplatin based on the organoid drug sensitivity testing (**Figure 5C**), this regimen was continued. On May 9, 2025, the CT scan showed SD, but PD of lung metastases occurred during a re-examination on July 1, 2025. Therefore, the treatment regimen was switched to paclitaxel micelle plus S-1 still shown sensitive in the drug sensitivity testing for 3 cycles, and SD was achieved.

Pat09 was diagnosed with right lung adenocarcinoma staged TxNxM1b (stage IVA), accompanied by bone metastasis, mediastinal lymph node metastasis, and liver metastasis. On September 23, 2024, percutaneous lung biopsy was performed for pathological examination, which confirmed lung adenocarcinoma (**Figure 5D**). On November 14, 2024, local sacral radiotherapy was administered. A CT scan on November 28, 2024, showed slight progression of pulmonary metastatic lesions compared with the previous examination. After treatment with pemetrexed disodium for one cycle, stereotactic body radiation therapy was delivered to the right lung tumor. On January 20, 2025, the CT scan revealed a reduction in the size of pulmonary lesions, thus pemetrexed was continued. On March 17, 2025, the CT scan showed new patchy hypodense lesions in the liver (**Figure 5E**). Percutaneous liver biopsy indicated metastatic lung adenocarcinoma in the liver, suggesting PD. Meanwhile, the biopsy tissue was collected for organoid culture, during which vinorelbine was administered. On April 23, 2025, the patient was treated with lucanthone Sacituzumab Tirumotecan that was shown most sensitive among all the treatments detected by the PDTO-based drug sensitivity testing (**Figure 5F**). After 3 cycles of treatment, the patient achieved SD.

## 4 Discussion

Treatment resistance represents a primary hurdle impeding the efficient management of advanced solid tumors, arising from either intrinsic or acquired biological mechanisms (19, 20). Emerging studies have delineated the underlying drivers of this resistance, ranging from genomic alterations and activation of diverse signaling cascades to changes of the tumor microenvironment (TME) (21–23). While these findings shed light on how pharmacotherapies may modulate genomic landscapes in advanced solid tumors, there remains a lack of actionable evidence to guide patient stratification or forecast the treatment outcomes. Therefore, there is an urgent need for the models that accurately mimic the key features of advanced solid tumors and predict the responses to therapeutic interventions. In this study, we characterized the key culture strategies for the successful establishment of the organoids from solid tumors, and verified that PDTOs could partially recapitulate the histopathological features and proliferative profiles of matched original tumors. More importantly, PDTO-based drug sensitivity testing results exhibited a good concordance with clinical treatment outcomes, with a Kappa coefficient of 0.625 and an overall accuracy of 85.0%, which further confirmed the clinical translational potential of PDTOs in guiding personalized treatment for advanced solid tumors.

In our study, the establishment success rates of organoids across different cancer types ranged from 90.6% to 92.6%, with the overall success rate of 91.5%, which were superior to 75%-83% for pancreatic cancer (24), 74.6% for gastric cancer (25), >80% for breast cancer (26) and 75.7% for lung cancer (18) reported in previous studies. This may be partially attributed to the optimized culture strategies tailored to the biological characteristics of different sample sources. For tissue samples, refined primary cell isolation procedures effectively preserved cell viability and heterogeneity, while efficient tumor cell enrichment strategies significantly improved the culture success rate for malignant effusion samples with low cell abundance. Additionally, the standardized quality control system covering cell viability detection, microbial contamination screening and phenotypic validation ensured the reliability and stability of organoid models. Notably, histopathological identification further validated the capacity of organoids derived from multiple cancer types to retain and recapitulate the histopathological features and proliferative activity of their corresponding original tumors, laying a solid foundation for subsequent drug sensitivity testing and clinical application.

The primary objective of this study was to explore the potential of PDTOs as a companion diagnostic tool for personalized prediction of treatment responses in advanced solid tumors. Notably, all monotherapies and combination regimens applied in PDTO-based drug screening and clinical management are standard guideline-endorsed treatments for advanced solid tumors. Rather than replacing clinical guideline recommendations, PDTO-based drug screening stratifies therapeutic responses to these approved regimens on a patient-specific basis, enabling clinicians to prioritize optimal treatment options within established clinical workflows and improve treatment outcomes for drug-refractory cases. By comparing the previous treatment outcomes of advanced cancer patients with their organoid drug screening results, we found a good concordance between them, with the overall accuracy reaching 85.0%. Zu et al. evaluated the ability of gastric cancer organoid drug phenotypes to recapitulate the corresponding patient’s treatment response, and a concordance rate of 75.0% was identified (27). Leveraging a living organoid biobank, Yao et al. further demonstrated that the patients’ chemoradiation responses were highly consistent with those of the corresponding rectal cancer organoids (28), with an overall accuracy of 84.43%, which was further validated in follow-up studies (29, 30). For pancreatic cancer, where the overall efficacy of chemotherapy remains suboptimal, the screening of patient-specific sensitive drugs is critically important (31). Grossman et al. performed drug sensitivity testing on 12 pancreatic cancer organoids, and observed that different organoids exhibited distinct sensitivity to the same drug, and the same organoid also showed variable sensitivity to different chemotherapeutic agents (32). Meanwhile, the drug responses of organoids correlated with clinical responses in individual patients. These findings not only support the value of PDTOs in predicting treatment responses for advanced solid tumors, but also imply their potential utility as a companion diagnostic tool to guide clinical decision-making in this patient population. Importantly, for advanced solid tumors with multidrug resistance, such as the cholangiocarcinoma and lung adenocarcinoma cases reported in our study, organoid drug screening identified personalized sensitive agents for the patients, and SD was achieved through the subsequent treatment guided by the screening results. This not only alleviated tumor-associated symptoms but also prolonged disease control duration, thereby highlighting the unique value of PDTOs in overcoming multidrug resistance in advanced solid tumors.

This study has several limitations that need to be addressed. First, the clinical validation cohort had a relatively small sample size, and enrolled multiple heterogeneous solid tumors. These tumor types possess distinct biological characteristics, microenvironmental landscapes, and treatment responses, resulting in prominent inter-tumor heterogeneity. Pooled analysis of these diverse cancers may compromise statistical accuracy and confuse the interpretation of PDTO-clinical response concordance, reducing subtype-specific reference value. Subgroup analyses were not performed due to insufficient validation cases per subtype. Future large-scale, multicenter studies with stratified subgroup validations are warranted to further verify the clinical translational value of PDTOs in individualized tumor treatment. Second, the drug concentration used in *in vitro* drug sensitivity testing was based on the upper and lower limits of blood drug concentration, and the inconsistency between *in vitro* drug concentration and *in vivo* tumor tissue drug concentration may also lead to deviations in screening results. Third, although our data confirmed that the established PDTOs recapitulated the histological features and proliferative activity of primary tumors, this study lacked comprehensive IHC staining panels, paired genomic and transcriptomic profiling of tumors and matched organoids, as well as systematic multi-parametric evaluation for drug response. More comprehensive characterization of lineage-specific markers, tumor-associated molecules, genomes and transcriptomes, as well as multi-parametric functional readouts including apoptosis staining, live/dead staining, and image-based organoid size quantification, will be incorporated in future studies. Additionally, the current PDTO models mainly recapitulate the properties of tumor parenchymal cells, but lack of immune cells, stromal cells and other components of the TME may compromise their predictive capability for immunotherapy. Recently, PDTOs models have been integrated into a technical platform that assesses individualized immunotherapy responses through the co-culture of tumor organoids with peripheral blood lymphocytes (33). In the future, the optimization of organoid-immune cell co-culture systems, such as the incorporation of tissue-resident immune subsets (34) and vascularized microenvironments (35), will further enhance the translational potential of this model for immunotherapy screening.

## 5 Conclusions

Our study successfully established a high-quality PDTO biobank with high culture success rates, and demonstrated that PDTOs exhibited favorable potential for predicting treatment responses in patients with advanced solid tumors, suggesting that PDTOs may serve as a promising auxiliary companion diagnostic tool to inform personalized cancer therapy and assist in evaluating multidrug resistance for advanced solid tumors. However, the small clinical validation cohort in this study limits the robustness and generalizability of the findings. Future large-scale, multicenter studies with stratified subgroup validations are warranted to further verify the clinical translational value of PDTOs in individualized tumor treatment.

## Data Availability

The raw data supporting the conclusions of this article will be made available by the authors, without undue reservation.

## Supplementary material

Supplementary Figure 1 Representative bright-field microscopic images of PDTOs established from all 23 patients in the drug-testing cohort. All microscopic images were acquired at ×100 magnification.
