# Supplementary figures and images for: From patient-derived tumor organoids to personalized cancer therapy: advancing treatment for advanced solid tumors
Source: Front Oncol. 2026 Jul 20;16:1880986. doi: 10.3389/fonc.2026.1880986 (PMC13429446; doi:10.3389/fonc.2026.1880986)

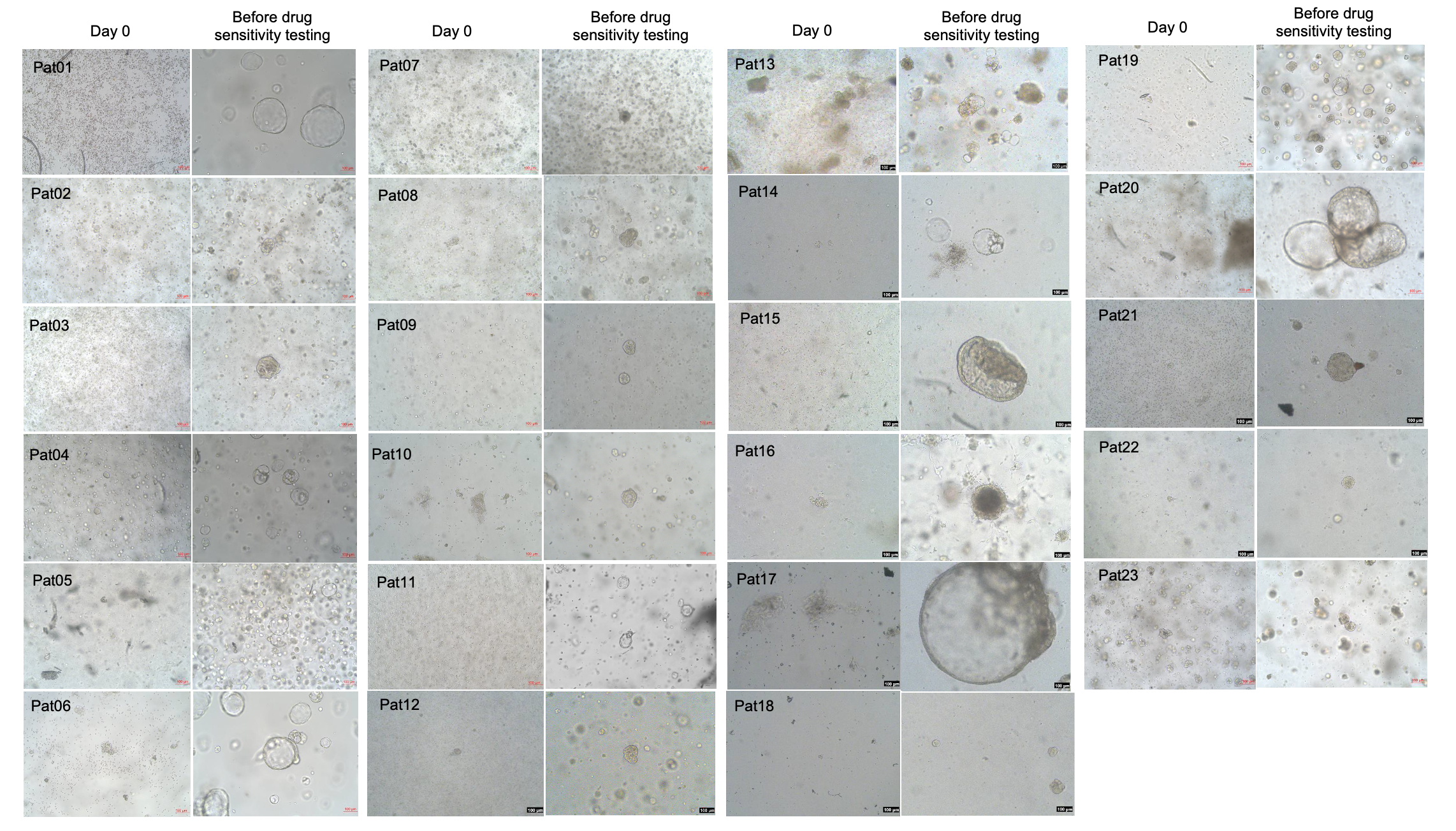

Supplement: Supplementary Figure 1 — Representative bright-field microscopic images of PDTOs established from all 23 patients in the drug-testing cohort. All microscopic images were acquired at ×100 magnification. [file Image1.tiff]
