# Supplementary material for: From patient-derived tumor organoids to personalized cancer therapy: advancing treatment for advanced solid tumors
Source: Front Oncol. 2026 Jul 20;16:1880986. doi: 10.3389/fonc.2026.1880986 (PMC13429446; doi:10.3389/fonc.2026.1880986)
Supplement: Supplementary file 2 [file Table1.docx]

**Supplementary Table 1.** The drug concentrations for organoid drug sensitivity testing

| Drug classification | Drug names | Cmax |
| --- | --- | --- |
| Chemotherapeutic agents | 5-fluorouracil | 5.00 × 10⁻⁶ M |
|  | Oxaliplatin | 2.00 × 10⁻⁶ M |
|  | Liposomal irinotecan | 1.00 × 10⁻⁸ M |
|  | Cisplatin | 1.00 × 10⁻⁵ M |
|  | Etoposide | 5.00 × 10⁻⁶ M |
|  | Dacarbazine | 5.00 × 10⁻⁵ M |
|  | Capecitabine | 5.00 × 10⁻⁶ M |
|  | Paclitaxel micelles | 5.00 × 10⁻⁶ M |
|  | Gemcitabine | 1.00 × 10⁻⁶ M |
|  | Calcium folinate | 2.00 × 10⁻⁴ M |
|  | Vinorelbine | 1.00 × 10⁻⁷ M |
|  | Lobaplatin | 1.00 × 10⁻⁵ M |
|  | Raltitrexed | 1.00 × 10⁻⁶ M |
|  | Thiotepa | 1.00 × 10⁻⁵ M |
|  | Pemetrexed | 2.00 × 10⁻⁴ M |
|  | Carboplatin | 4.00 × 10⁻⁵ M |
|  | Albumin-bound paclitaxel | 8.00 × 10⁻⁶ M |
|  | Lurbinectedin | 1.00 × 10⁻⁷ M |
|  | Teniposide | 5.00 × 10⁻⁵ M |
|  | Lomustine | 1.00 × 10⁻⁵ M |
|  | S-1 | 1.00 × 10⁻⁶ M |
|  | Trifluridine/tipiracil | 1.00 × 10⁻⁵ M |
|  | Eribulin | 1.00 × 10⁻⁷ M |
|  | Paclitaxel | 3.00 × 10⁻⁶ M |
| Targeted agents | Lenvatinib | 5.00 × 10⁻⁷ M |
|  | Anlotinib | 1.00 × 10⁻⁷ M |
|  | Surufatinib | 1.00 × 10⁻⁶ M |
|  | Fruquintinib | 5.00 × 10⁻⁷ M |
|  | Pazopanib | 5.00 × 10⁻⁵ M |
|  | Olaparib | 1.00 × 10⁻⁵ M |
|  | Fluzoparib | 1.00 × 10⁻⁵ M |
|  | Everolimus | 1.00 × 10⁻⁷ M |
|  | Exemestane | 5.00 × 10⁻⁸ M |
|  | Palbociclib | 1.00 × 10⁻⁷ M |
|  | Fulvestrant | 5.00 × 10⁻⁸ M |
|  | Chidamide | 1.00 × 10⁻⁷ M |
|  | Vorinostat | 5.00 × 10⁻⁶ M |
|  | Alpelisib | 5.00 × 10⁻⁶ M |
|  | Trametinib | 1.00 × 10⁻⁸ M |
|  | Temozolomide | 5.00 × 10⁻⁵ M |
|  | Celecoxib | 1.00 × 10⁻⁶ M |
|  | Hydroxychloroquine | 5.00 × 10⁻⁷ M |
| Monoclonal antibodies | Nimotuzumab | 0.005 mg/mL |
|  | Sacituzumab govitecan | 0.005 mg/mL |
|  | Cetuximab | 0.1 mg/mL |
|  | Trastuzumab deruxtecan | 0.1 mg/mL |
|  | Disitamab vedotin | 0.005 mg/mL |
